# Financial Toxicity in Patients With Breast Cancer Treated at Private Medicine Institutions in Brazil

**DOI:** 10.1002/cam4.71664

**Published:** 2026-03-10

**Authors:** Aline Cristini Vieira, Matheus Costa e Silva, Cristiano De Padua Souza, Luciana Castro Garcia Landeiro, Max Senna Mano

**Affiliations:** ^1^ Oncology Department Hospital Sírio‐Libanês São Paulo Brazil; ^2^ Bachelor's Degree in Biomedical Informatics, Grupo Oncoclínicas São Paulo Brazil; ^3^ Oncology Department Hospital do Cancer de Barretos Barretos Brazil; ^4^ Oncology Department, Nucleo de Oncologia Da Bahia Grupo Oncoclinicas Salvador Brazil

## Abstract

Financial toxicity (FT) refers to the negative impact of the financial burden of cancer diagnosis and treatment on patients' well‐being. With rising global costs of oncologic therapies, diagnostics, and supportive care, FT has become a key concern in cancer care, influencing adherence, quality of life, and outcomes. Breast cancer, given its high prevalence and socioeconomic implications, is notably associated with FT. Beyond direct medical expenses, patients often face income loss and out‐of‐pocket costs for supportive care, which can significantly affect their therapeutic trajectory. Addressing FT is thus essential to ensure equitable cancer care. A 34‐item questionnaire, including the Comprehensive Score for Financial Toxicity (COST) tool, was administered to breast cancer patients treated at three private institutions in Brazil. Data were analyzed using descriptive statistics, linear regression, and ANOVA (*p* ≤ 0.05). Among 912 participants 85.1% self‐identified as White. Most were married or in a stable union (67.8%), and 50.9% held a postgraduate degree. A majority (84.2%) had early‐stage disease, 54.6% had been diagnosed within 2 years prior to the study, and 92.1% reported having health insurance. Despite this, 36.6% experienced a reduction in their standard of living following treatment, and 15.6% reported having to sell assets or property. The mean COST score was 24.2 (SD = 8.6; median = 25.0). Factors significantly associated with greater financial toxicity (lower COST scores) included age below 40 years, lack of health insurance, unemployment or medical leave, and household income below 10,000 BRL per month. These findings highlight the considerable financial burden experienced by breast cancer patients, even in the private healthcare system, and underscore the need for strategies to mitigate FT in oncology care.

## 1 Introduction

Financial toxicity (FT) is increasingly recognized as a significant adverse outcome of cancer care. It refers to the economic burden experienced by patients as a result of the disease and its treatments, encompassing both objective financial hardship and subjective financial distress related to cancer care [1, 2, 3, 4, 5]. Given that breast cancer is the most commonly diagnosed cancer type among women both globally and in Brazil [6, 7], understanding the factors associated with FT in this population is essential. Advances in diagnosis and treatment have contributed to improved survival; however, these advances often come with a high financial burden for patients. In low‐ and middle‐income countries like Brazil, where access to private healthcare may result in out‐of‐pocket expenses even for insured patients, the risk of FT may be even greater. Therefore, it is crucial to assess additional costs, such as copayments for treatment, supportive care, and nonmedical or indirect costs (e.g., travel, caregiver time, and lost productivity), which may impose a substantial financial burden even on patients with health insurance coverage [8, 9]. Furthermore, evidence from high‐income countries indicates that FT is associated with shorter survival and reduced quality of life, and increased mortality risk [10, 11]. Brazil has both a public and private health insurance system. However, only 24.5% of the Brazilian population is covered by private insurance [12]. A substantial proportion of these beneficiaries rely on employer support to finance their plans, 46.2% pay directly, while 45.4% have costs partially or fully covered by their employers [13].

This study examines the burden and determinants of FT among Brazilian breast cancer patients treated at leading private healthcare institutions.

## 2 Methods

This study included patients diagnosed with breast cancer between 2016 and 2020 who received treatment at one of three leading private cancer centers in Brazil, located in the South, Southeast, and Northeast regions of the country. Eligible participants were female, aged 18 years or older, with a histologically confirmed diagnosis of breast cancer, and had undergone treatment that included chemotherapy, targeted therapy, hormone therapy, surgery, and/or radiotherapy.

This study received approval from the Institutional Ethics Committees of all three participating institutions: the Research and Teaching Institute of Hospital Sírio‐Libanês; the Research Ethics Committee of the Hospital de Clínicas, Federal University of Paraná, on behalf of Oncoclínicas Curitiba; and the Prof. Dr. Celso Figueirôa Research Ethics Committee of Hospital Santa Izabel, representing the Oncology Center of Bahia. Ethical approval was obtained prior to the commencement of the study, and all procedures were conducted in accordance with the Declaration of Helsinki and relevant national guidelines. Electronic informed consent was obtained from all participants prior to their inclusion in the study, as approved by the Ethics Committees. The consent form was presented at the beginning of the online questionnaire, and only participants who agreed to participate were able to proceed. Those who did not provide consent were unable to access or complete the questionnaire.

A questionnaire containing 34 questions on demographic and socioeconomic background and the COST score was applied. To assess FT, we utilized the Comprehensive Score for Financial Toxicity (COST), a validated tool that has proven effective in measuring the financial burden on patients with cancer [14]. The COST score is an 11‐item patient‐reported outcomes measure used to evaluate FT. Scores range from 0 to 4 for every item; thus, the total COST score can range from 0 to 44, with a lower score representing a greater degree of FT and vice versa. The FT grading system was divided into four levels based on the COST score: G0 (no FT) with a score of ≥ 26; G1 (mild FT) with a score between ≥ 14 and 26; G2 (moderate FT) with a score between > 0 and 14; and G3 (severe FT) with a score of 0 [15].

We used the methodology of the Functional Assessment of Chronic Illness Therapy (FACIT) to translate the original COST questionnaire from English into Portuguese [16]. Two researchers independently translated the questionnaire into Portuguese and subsequently integrated their versions. A back‐translation into English was then performed and reviewed by a native English speaker to ensure fidelity to the original version. Additionally, other co‐authors reviewed the final Portuguese version. The questionnaire was validated calculating the Cronbach's alpha test which was 0.88, indicating a good internal consistency [17].

### 2.1 Statistical Analyses

Patient characteristics are summarized using descriptive statistics. The statistical analysis was initially conducted using various summary measures such as mean, median, minimum and maximum values, standard deviation, absolute and relative frequencies (percentage), along with the unidimensional scatter plot.

Inferential analyses included Analysis of Variance (ANOVA) with one fixed factor and with seven fixed factors as appropriate [18]. A significance level (alpha) of 5% was applied to all statistical tests. Data were entered into Excel for storage and analyzed using the IBM‐SPSS Statistics, version 24.

The correlation coefficient (*β*) and corresponding 95% confidence intervals (CI) were calculated. Associations between sociodemographic and outpatient variables and the COST score [14] were evaluated.

## 3 Results

The sample consisted of 912 patients, of whom 776 (85.1%) self‐identified as White, 58 (6.4%) as Black, 14 (1.5%) as Asian, and 64 (7.0%) as belonging to other racial groups. At the time of diagnosis, approximately 25.0% of patients were under 40 years of age, 36.4% were between 40 and 50, and 29.2% were between 51 and 64. At the time of data collection, most patients (67.8%) were married or in a stable union, and 50.9% held postgraduate degrees, including specialization, master's, doctoral, or PhD qualifications (Table 1). Lifestyle characteristics revealed that most patients (94.4%) were non‐smokers, and approximately 395 (43.3%) reported no alcohol consumption.

**TABLE 1:** General characteristics of patients.

Category	Subcategory	Count	Percentage
Race (*n* = 912)	Asian	14	1.5
	White	776	85.1
	Black	58	6.4
	Other	64	7.0
Current age range (*n* = 912)	Less than 40 years	144	15.8
	40–50 years	304	33.3
	51–64 years	320	35.1
	65–75 years	109	12.0
	More than 75 years	35	3.8
Age at diagnosis (*n* = 911)	Less than 40 years	228	25.0
	40–50 years	332	36.4
	51–64 years	266	29.2
	65–75 years	68	7.5
	More than 75 years	17	1.9
Current marital status (*n* = 909)	Married/stable union	616	67.8
	Divorced/separated	140	15.4
	Single/never married	99	10.9
	Widowed	54	5.9
Marital status at diagnosis	Married/stable union	649	71.2
	Divorced/separated	113	12.4
	Single/never married	111	12.2
	Widowed	39	4.3
Change in marital status after diagnosis (*n* = 912)	No	829	90.9
	Yes	83	9.1
Educational level (*n* = 912)	Less than high school	22	2.4
	High school graduate	102	11.2
	College/university	324	35.5
	Postgraduate	464	50.9
Family income (*n* = 912)	Less than 10,000 reais/month	306	33.6
	10,000–20,000 reais/month	324	35.5
	20,000–50,000 reais/month	215	23.6
	More than 50,000 reais/month	67	7.3
Current employment status (*n* = 912)	Full‐time employment	310	34.0
	Part‐time employment	81	8.9
	Self‐employed	151	16.6
	Other	13	1.4
	Homemaker	82	9.0
	Receiving sickness benefits/absent from work	46	5.0
	Retired	179	19.6
	Unemployed	50	5.5
Current smoking habit (*n* = 912)	Non‐smoker	861	94.4
	Quit smoking within the last 6 months	16	1.8
	Smoker	35	3.8
Current alcohol consumption habit (*n* = 912)	Does not consume alcoholic beverages	395	43.3
	Yes, more than once a week	167	18.3
	Yes, less than once a week	350	38.4
Since the diagnosis of breast cancer, have you	No change in work routine	382	41.9
	Increased workload	34	3.7
	Reduced workload	188	20.6
	Stopped working by choice	69	7.6
	Unable to return to work	71	7.8
	Other/not applicable	168	18.4
Time since diagnosis	2 years or less	498	54.6
	2–5 years	317	34.8
	More than 5 years	97	10.6
Hospitalizations in the last 2 years due to breast cancer	2 or fewer hospitalizations	725	79.5
	3 or more hospitalizations	102	11.2
	Don't know	85	9.3
Visits to the emergency room/service due to your illness	2 or fewer visits to the ER	679	74.5
	3 or more visits to the ER	110	12.1
	Don't know	123	13.5
Stage of disease at diagnosis	Initial disease (localized)	768	84.2
	Metastatic/advanced stage	122	13.4
	Don't know	22	2.4
Type of health insurance	Private health insurance (individual)	279	30.6
	Health insurance by individual adherence scheme (linked to an association, union, or professional entity)	135	14.8
	Corporate health insurance (business)	426	46.7
	Other	28	3.1
	Does not have health insurance	44	4.8
How do you consider your health?	Excellent	361	39.6
	Good	440	48.2
	Fair	96	10.5
	Poor	15	1.6
How do you describe your psychological state in relation to your treatment?	Optimistic/confident	679	74.5
	Insecure	152	16.7
	Depressed/pessimistic	53	5.8
	Indifferent	16	1.8
	Other	12	1.3

Most patients (84.2%) had early‐stage breast cancer. Slightly more than half of the participants (54.6%) had been diagnosed with breast cancer within 2 years prior to the study. The majority (92.1%) reported having some form of health insurance and rated their health status as either excellent or good (87.8%). Additionally, 679 patients (74.5%) reported feeling optimistic or confident when reflecting on their psychological status in relation to their treatment. Table 2 summarize patient's perceptions of their current quality of life and the burden associated with out‐of‐pocket expenses for cancer care.

**TABLE 2:** Impressions about the standard of living in light of patients' disease diagnosis.

Aspect	Category	*N*	Percentage
Would you like your doctor to discuss the costs (beyond those covered by your insurance) of treatment with you?	No	172	18.9
	Don't know	142	15.6
	Yes	598	65.6
Do you feel that you had to reduce your standard of living since your cancer diagnosis?	No	554	60.7
	Don't know	24	2.6
	Yes	334	36.6
Have you had to dispose of assets/property due to your cancer treatment?	No	770	84.4
	Yes	142	15.6
If you had to dispose of assets and property, what is the estimated amount of reduction in assets/property?	Less than 25%	102	11.2
	25%–50%	45	4.9
	50%–75%	13	1.4
	75%–100%	4	0.4
	Did not have to dispose of assets/property	742	81.4
	Did not respond	6	0.7

A total of 83 patients (9.1%) experienced a change in marital status during the study period. Regarding socioeconomic status, 630 patients (69.1%) reported a monthly household income of up to 20,000 reais (approximately 4000 USD, based on 2023 exchange rates), and 391 (42.9%) were employed either full‐time or part‐time.

Five hundred and ninety‐eight (65.6%) patients expressed a desire for their physicians to address the financial implications of treatment during clinical consultations. As summarized in Table 1, 382 patients (41.9%) reported no change in their work routine since their breast cancer diagnosis. Furthermore, 333 patients (36.6%) reported a reduced standard of living following treatment, and 142 (15.6%) were forced to sell assets or property after diagnosis. Among them, only 6.7% had to liquidate 25% or more of their assets (Table 2).

**GRAPH 1:**
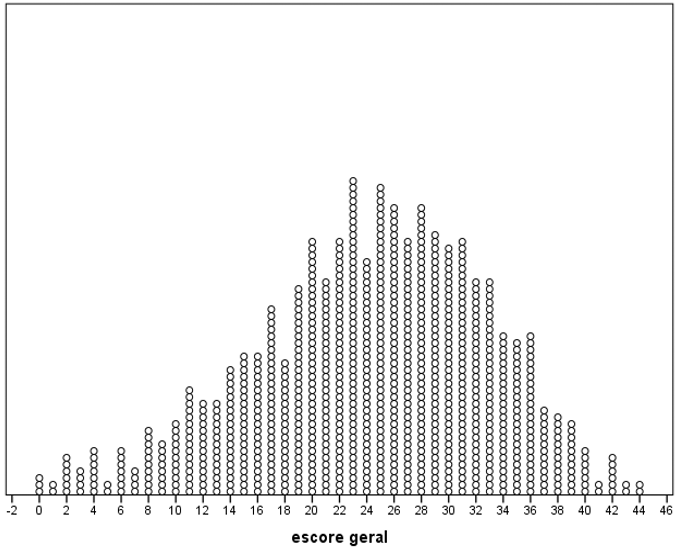
Unidimensional scatter plot of the overall scores of patients in the COST questionnaire.

The overall scores of patients on the COST questionnaire were analyzed for a sample of 912 participants. The mean score was 24.2, with a median score of 25.0, indicating a central tendency around the middle of the scoring range (Graph 1). The scores ranged from a minimum of 0.0 to a maximum of 44.0, highlighting variability in patient responses. The standard deviation of the scores was 8.6, reflecting a moderate spread of values around the mean (Table 3).

**TABLE 3:** Summary measure of the overall score of patients in the COST questionnaire.

Measure	Value
Sample size (*n*)	912
Mean	24.2
Median	25.0
Minimum–maximum	0.0–44.0
Standard deviation	8.6

Based on the inferential results from the univariate analyses, patients under the age of 40 exhibited lower COST scores compared to other age groups (*p* < 0.001) (Tables 4 and 5). Similarly, patients without health insurance had significantly lower scores than those with private coverage, including individual plans, plans through membership organizations, and corporate insurance (*p* < 0.001) (Detailed multiple‐comparison results are provided in the Appendix S1.), and was significantly associated with financial toxicity (*p* < 0.001). Patients on and those who were unemployed had the lowest COST scores. Retired individuals had significantly higher scores than both groups, as well as compared to self‐employed patients (Appendix S2).

**TABLE 4:** Summary measures of the overall scores of patients, according to some characteristics.

Characteristic	Category	*n*	Mean	Median	SD	*p*
Age at diagnosis	Less than 40 years	228	21.5	23.0	9.0	< 0.001
	40–50 years	332	24.0	25.0	8.4	
	51–64 years	266	26.0	27.0	8.1	
	65–75 years	68	26.4	27.0	8.1	
	More than 75 years	17	27.9	24.0	7.8	
Marital status at diagnosis	Married/stable union	649	24.2	25.0	8.8	0.663
	Divorced/separated	113	24.4	25.0	7.7	
	Single/never married	111	23.6	24.0	8.7	
	Widowed	39	25.6	27.0	8.7	
Type of health insurance	Private health insurance (individual)	279	25.0	26.0	8.5	0.003
	Health insurance by individual adherence scheme	135	25.1	25.0	8.3	
	Corporate health insurance (business)	426	23.8	25.0	8.4	
	Other	28	24.6	25.5	10.1	
	Does not have health insurance	44	19.9	21.5	10.3	
Current employment status	Works full‐time	310	25.7	26.0	7.4	< 0.001
	Works part‐time	81	26.0	25.0	7.2	
	Self‐employed	151	23.5	25.0	8.3	
	Other	13	27.8	28.0	8.5	
	Homemaker	82	23.7	24.0	9.0	
	Receiving sickness/disability benefits	46	17.5	20.0	9.1	
	Retired	179	26.2	27.0	8.5	
	Unemployed	50	13.3	13.0	6.9	
Race	Asian	14	26.9	27.5	7.4	< 0.001
	White	776	24.8	25.0	8.4	
	Black	58	18.8	19.0	9.6	
	Other	64	21.7	23.0	8.0	
Health status	Excellent	361	27.0	28.0	7.8	< 0.001
	Good	440	23.6	24.0	8.1	
	Fair	96	17.6	18.0	9.2	
	Poor	15	16.5	17.0	7.8	
Family income	Less than 10,000 reais/month	306	19.1	20.0	8.4	< 0.001
	10,000–20,000 reais/month	324	25.3	26.0	7.6	
	20,000–50,000 reais/month	215	27.8	28.0	6.9	
	More than 50,000 reais/month	67	31.0	32.0	7.0	

*Note:* (a) Minimum value, (b) maximum value, (c) standard deviation, (d) analysis of variance (ANOVA) with one fixed factor (univariate analysis).

**TABLE 5:** Results of multiple comparisons (Tukey method) for age groups at diagnosis.

Conclusion	*p*
Less than 40 years < 40–50 years	0.005
Less than 40 years < 51–64 years	< 0.001
Less than 40 years < 65–75 years	< 0.001
Less than 40 years < More than 75 years	0.022
40–50 years < 51–64 years	0.037
40–50 years = 65–75 years	0.215
40–50 years = More than 75 years	0.347
51–64 years = 65–75 years	0.997
51–64 years = More than 75 years	0.896
65–75 years = More than 75 years	0.965

Employment status was significantly associated with financial toxicity (*p* < 0.001). Patients on sick leave and those who were unemployed had the lowest COST scores. Retired individuals had significantly higher scores than both groups, as well as compared to self‐employed patients.

Regarding race, Black patients had lower scores compared to White and Asian patients, while individuals identifying as mixed‐race (pardo), Indigenous, or other non‐specified groups (classified as “other”) also scored lower than White patients (*p* < 0.001) (Appendix S3). Patients who rated their health as excellent demonstrated significantly higher scores compared to all other self‐rated health categories (*p* < 0.001) (Appendix S4). Lastly, patients with a household income below 10,000 reais per month reported lower scores than all other income groups (*p* < 0.001) (Appendix S5).

## 4 Discussion

To our knowledge, this is the first study evaluating factors associated with FT in patients with cancer in private institutions in Brazil. FT can exert a direct influence on the quality of cancer care, potentially leading patients to skip prescribed medications to save costs, employ expired medications, sidestep comprehensive multidisciplinary assessments (e.g., psychotherapy, dental care, physiotherapy), and forgo routine check‐up appointments [19]. A large American study found that patients with cancer were 2.65 times more likely to file for bankruptcy compared to individuals without cancer. Younger patients, in particular, were two to five times more likely to declare bankruptcy than those aged 65 or older [20]. In another study, patients with cancer were 71% more likely to experience financial hardships—including foreclosures, tax liens, third‐party collections, delinquent mortgage payments, and repossessions—compared to similar individuals without cancer [21]. Additionally, patients who filed for bankruptcy had a 79% higher risk of mortality [10].

By employing the COST questionnaire, our study unveiled that more than one‐third of patients with breast cancer treated at Brazilian private institutions reported adopting strategies to reduce expenses and lower their standard of living, even though the study population predominantly had health insurance and access to high‐quality cancer care. These findings underscore the significant prevalence of FT among patients with breast cancer in Brazil, even within the context of a relatively privileged population with access to private healthcare services.

The majority of patients (65.6%) expressed a desire for their physician to discuss treatment‐related costs with them, which is consistent with findings reported in the literature [22, 23, 24]. However, cost communication in Brazil faces several systemic barriers, including limited transparency of health system and insurance costs, lack of structured training for oncologists in financial communication, time constraints in routine consultations, and cultural reluctance from both patients and physicians to address financial issues. To address this gap, potential strategies include the integration of financial navigators into oncology teams, the development of standardized tools that facilitate patient–physician conversations about affordability, and the incorporation of financial literacy and cost communication into oncology curricula. Such initiatives may promote more patient‐centered care and mitigate the adverse impact of financial toxicity.

Several studies have consistently identified associations between COST scores and a range of factors [9, 25], including race, employment status, frequency of hospitalizations, age, marital status, time since diagnosis, income, and out‐of‐pocket medical expenses [26].

The median COST score in our study was 24.2 which is graded as mild financial toxicity. This value is slightly higher than that reported in a Chinese study involving patients with breast cancer, which found a median score of 22.5 [27]. Similarly, a Japanese study that included patients with various tumor types reported a median score of 21.0 [28]. In contrast, studies focusing on specific cancer types showed more variability: patients with gynecologic neoplasms had a median COST score of 20.5 [29], while those with multiple myeloma and lung cancer demonstrated higher median scores of 27.0 [25, 30]. COST scores vary notably across different cancer types and healthcare systems, reflecting distinct patterns of financial burden. In our study with breast cancer patients, the median score was 24.2, indicative of mild financial toxicity. Lower scores have been observed in patients with gynecologic cancers (median: 20.5) [29] and in studies from China and Japan involving breast cancer and mixed tumor types, with medians of 22.5 and 21.0, respectively [27, 28]. Conversely, higher scores were reported among patients with multiple myeloma and lung cancer, both showing median COST scores of 27.0 [25, 30]. These differences may be attributed to variations in disease course, treatment complexity, duration of care, and access to supportive resources.

Although the median COST score in our study indicated mild financial toxicity, the high proportion of patients reporting a reduction in standard of living (36.6%) and selling assets or property (15.6%) highlights a possible discrepancy between standardized scoring and the lived experience of patients. This may reflect several factors. First, cultural and social desirability biases may lead Brazilian patients to underreport financial difficulties in structured questionnaires. Second, although validated, the COST instrument may not fully capture indirect and catastrophic costs, which are particularly relevant in middle‐income countries. Third, patients with private health insurance may still face hidden out‐of‐pocket expenses (such as copayments, transportation, or caregiver costs) that significantly affect household finances but are not directly reflected in the COST score. These findings suggest that while COST is a valuable tool, further cross‐cultural validation and methodological adaptation may be needed to better capture the multidimensional aspects of financial toxicity in Brazil.

Our research revealed that among Brazilian breast cancer patients, the COST score was significantly associated with several key factors, including age under 40, non‐white ethnicity, lower household savings, unemployment, work leave, reduced or discontinued work due to cancer, poor health status, and a decreased standard of living since the cancer diagnosis. These findings shed light on the multifaceted nature of FT among patients with breast cancer in Brazil, highlighting its connection with various socio‐demographic and health‐related factors.

Financial toxicity is particularly pronounced among younger patients, partly because they are often diagnosed with more aggressive tumors and have poorer prognoses, which demand intensive and costly treatments [31]. In our research, 25.0% of patients were under the age of 40 at the time of diagnosis. This is relevant because, typically, they have lower incomes and relatively modest savings, may have higher expenses tied to their children's needs and the pursuit of their burgeoning professional careers [32]. A cancer diagnosis often triggers a cascade of challenges, including diminished career prospects, lost workdays, and changes in job roles. These financial disruptions extend beyond the treatment phase, often persisting for years and influencing critical life decisions, with a significant risk of bankruptcy [5, 20].

Marital status is considered a prognostic factor for survival in patients who experience breast cancer [33]. Unfortunately, changes in marital status following a cancer diagnosis are not uncommon and may contribute significantly to financial distress among affected women [34]. In Brazil, the AMAZONA III study reported that 5.8% of married or cohabiting women with breast cancer underwent divorce or separation within 2 years of diagnosis [35]. In our study, approximately 10% of the women experienced a change in marital status; in contrast to its prognostic relevance, marital status did not show a significant association with FT in this study.

Conversely, unemployed patients and those on medical leave reported a significantly greater financial burden, primarily due to prolonged absence from the workforce and the resulting loss or reduction of income. When coupled with ongoing medical expenses, this financial strain places patients in a state of heightened economic vulnerability. In our study, these individuals experienced significantly higher levels of financial toxicity compared to others. These findings are consistent with previous research demonstrating that the inability to remain economically active during cancer treatment is a major contributor to FT [36, 37].

Additionally, women with breast cancer often face significant challenges when re‐entering the workforce after treatment. In our cohort, 7.8% were unable to return to work following their diagnosis. The process of professional reintegration is frequently complex and the reallocation process in this context is often complex, marked by physical, psychological, and social barriers. These findings underscore the need for supportive policies and targeted interventions aimed at facilitating workforce reentry and mitigating long‐term economic hardship [36, 37].

In addition, the cross‐sectional and observational design of our study precludes causal inference; thus, associations between employment status, health insurance, income, and financial toxicity should not be interpreted as directional. For example, while unemployment and work leave were significantly associated with higher financial toxicity, the temporal sequence cannot be determined in our analysis. Longitudinal studies in Brazil will be essential to clarify whether these socioeconomic disruptions precede financial toxicity or result from it, as well as to capture changes in financial burden over time. Moreover, many participants were still in the early stages of treatment, with short follow‐up, which may have limited the ability to capture the full extent of financial toxicity. Potential bias may also have arisen from patients' self‐reported responses due to varied interpretations of questionnaire items. Finally, our study population was composed exclusively of patients treated at private institutions, who likely have a higher socioeconomic status than the national average, which may limit the generalizability of the findings. This may also help explain the relatively low return‐to‐work rate in our sample (7.8%). Previous publications—including those focused on or including patients treated in the Brazilian public health system (SUS)—have consistently reported lower return‐to‐work rates among Brazilian women with breast cancer, highlighting persistent barriers to professional reintegration. Approximately 32% to 40% of patients had not returned to work even 2 years after diagnosis [38]. In this context, financial toxicity may be even more pronounced in populations treated in the public healthcare system, reinforcing the need for a broader, more inclusive national study currently underway.

Despite these limitations, the present analysis identified key factors associated with financial toxicity among Brazilian patients with breast cancer, offering valuable insights for cancer policy and healthcare planning. Beyond individual‐level factors, our findings have broader implications for health policy in Brazil. Recognizing financial toxicity as a critical dimension of cancer care quality, concrete measures are needed to mitigate its impact. Potential strategies include implementing financial navigation programs within oncology centers to guide patients through insurance coverage and out‐of‐pocket costs, developing income support policies for patients and caregivers affected by work disruptions, and establishing legal protections to safeguard employment rights during cancer treatment. Expanding insurance coverage to indirect costs, such as transportation and caregiver support, and strengthening social assistance programs are also essential to reduce the risk of catastrophic health expenditures. These initiatives, combined with greater cost transparency and improved physician–patient communication, could significantly lessen the burden of financial toxicity and promote more equitable cancer care. Furthermore, incorporating financial toxicity screening and support into survivorship care models is urgently needed. Finally, future research comparing public and private healthcare settings will be crucial to guide the development of equitable, targeted strategies to reduce the economic burden of cancer across diverse populations in Brazil.

## Author Contributions

Aline Cristini Vieira: conceptualization, data curation, formal analysis, visualization, writing – original draft, writing – review and editing. Mateus Costa e Silva: statistical analyses. Cristiano De Padua Souza: visualization, review and editing. Luciana Landeiro: review and editing. Max Senna Mano: conceptualization, visualization, review, and editing. All authors played an important role in interpreting the results; drafted or revised the manuscript; approved the final version; and agreed to be accountable for all aspects of the work in ensuring that questions related to the accuracy or integrity of any part of the work are appropriately investigated and resolved.

## Funding

The authors have nothing to report.

## Conflicts of Interest

The authors declare no conflicts of interest.

## Supporting information


**Data S1:** cam471664‐sup‐0001‐DataS1.docx.

## Data Availability

The data that supports the findings of this study are available in the Supporting Information of this article.

## References

[1] S. Y. Zafar , J. M. Peppercorn , D. Schrag , et al., “The Financial Toxicity of Cancer Treatment: A Pilot Study Assessing Out‐of‐Pocket Expenses and the Insured Cancer Patient's Experience,” Oncologist 18, no. 4 (2013): 381–390. 23442307 10.1634/theoncologist.2012-0279 PMC3639525

[2] S. Y. Zafar and A. P. Abernethy , “Financial Toxicity, Part I: A New Name for a Growing Problem,” Oncology 27 (2013): 80–149. 23530397 PMC4523887

[3] A. M. Gilligan , D. S. Alberts , D. J. Roe , and G. H. Skrepnek , “Death or Debt? National Estimates of Financial Toxicity in Persons With Newly‐Diagnosed Cancer,” American Journal of Medicine 131, no. 10 (2018): 1187–1199.e5. 29906429 10.1016/j.amjmed.2018.05.020

[4] A. J. Davidoff , M. Erten , T. Shaffer , et al., “Out‐of‐Pocket Health Care Expenditure Burden for Medicare Beneficiaries With Cancer,” Cancer 119 (2013): 1257–1265, 10.1002/cncr.27848. 23225522

[5] E. C. Dowling , N. Chawla , L. P. Forsythe , et al., “Lost Productivity and Burden of Illness in Cancer Survivors With and Without Other Chronic Conditions,” Cancer 119 (2013): 3393–3401, 10.1002/cncr.28214. 23794146 PMC5861717

[6] “Breast Cancer Now Most Common Form of Cancer: WHO Taking Action,” (2025), https://www.who.int/news/item/03‐02‐2021‐breast‐cancer‐now‐most‐common‐form‐of‐cancer‐who‐taking‐action.

[7] Ministério da Saúde , “Câncer de Mama,” (2025), https://www.gov.br/saude/pt‐br/assuntos/saude‐de‐a‐a‐z/c/cancer‐de‐mama/cancer‐de‐mama.

[8] H. Sung , J. Ferlay , R. L. Siegel , et al., “Global Cancer Statistics 2020: GLOBOCAN Estimates of Incidence and Mortality Worldwide for 36 Cancers in 185 Countries,” CA: A Cancer Journal for Clinicians 71 (2021): 209–249, 10.3322/caac.21660. 33538338

[9] L. G. Gordon , K. M. D. Merollini , A. Lowe , and R. J. Chan , “A Systematic Review of Financial Toxicity Among Cancer Survivors: We Can't Pay the Co‐Pay,” Patient 10, no. 3 (2017): 295–309. 27798816 10.1007/s40271-016-0204-x

[10] S. D. Ramsey , A. Bansal , C. R. Fedorenko , et al., “Financial Insolvency as a Risk Factor for Early Mortality Among Patients With Cancer,” Journal of Clinical Oncology 34, no. 9 (2016): 980–986. 26811521 10.1200/JCO.2015.64.6620 PMC4933128

[11] R. J. Chan , L. Gordon , S. Y. Zafar , and C. Miaskowski , “Financial Toxicity and Symptom Burden: What Is the Big Deal?,” Supportive Care in Cancer 26, no. 5 (2018): 1357–1359, 10.1007/s00520-018-4092-6. 29435714

[12] “Cresce o Número de Brasileiros Com Acesso a Plano de Saúde,” (2021), Saúde Business, https://www.saudebusiness.com/mercado/cresce‐o‐numero‐de‐brasileiros‐com‐acesso‐plano‐de‐saude.

[13] Agência Brasil , “IBGE: 59,7 Milhões de Pessoas Tinham Plano de Saúde em 2019,” (2020), https://agenciabrasil.ebc.com.br/saude/noticia/2020‐09/pesquisa‐diz‐que‐597‐milhoes‐de‐pessoas‐tinham‐plano‐de‐saude‐em‐2019.

[14] J. A. de Souza , B. J. Yap , K. Wroblewski , et al., “Measuring Financial Toxicity as a Clinically Relevant Patient‐Reported Outcome: The Validation of the COmprehensive Score for Financial Toxicity (COST): Measuring Financial Toxicity,” Cancer 123, no. 3 (2017): 476–484, 10.1002/cncr.30369. 27716900 PMC5298039

[15] J. A. De Souza , K. Wroblewski , E. Proussaloglou , L. Nicholson , A. Hantel , and Y. Wang , “Validation of a Financial Toxicity (FT) Grading System,” JCO 35, no. 15_suppl (2017): 6615, 10.1200/JCO.2017.35.15_suppl.6615.

[16] S. L. Eremenco , D. Cella , and B. J. Arnold , “A Comprehensive Method for the Translation and Cross‐Cultural Validation of Health Status Questionnaires,” Evaluation & the Health Professions 28 (2005): 212–232, 10.1177/0163278705275342. 15851774

[17] L. J. Cronbach , “Coefficient Alpha and the Internal Structure of Tests,” Psychometrika 16, no. 3 (1951): 297–334, 10.1007/BF02310555.

[18] J. Neter , Applied Linear Statistical Models, 4th ed. (Irwin, 1996).

[19] T. G. Knight , A. M. Deal , S. B. Dusetzina , et al., “Financial Toxicity in Adults With Cancer: Adverse Outcomes and Noncompliance,” Journal of Oncology Practice (2018), 10.1200/JOP.18.00120. 30355027

[20] S. D. Ramsey , D. K. Blough , A. C. Kirchhoff , et al., “Washington Cancer Patients Found to be at Greater Risk for Bankruptcy Than People Without a Cancer Diagnosis,” Health Aff (Millwood) 32, no. 6 (2013): 1143–1152. 23676531 10.1377/hlthaff.2012.1263 PMC4240626

[21] V. Shankaran , L. Li , C. Fedorenko , et al., “Risk of Adverse Financial Events in Patients With Cancer: Evidence From a Novel Linkage Between Cancer Registry and Credit Records,” Journal of Clinical Oncology 40 (2022): 884–891, 10.1200/JCO.21.01636. 34995125

[22] A. J. Bullock , E. W. Hofstatter , M. L. Yushak , and M. K. Buss , “Understanding Patients' Attitudes Toward Communication About the Cost of Cancer Care,” Journal of the Pancreas: JOP 8, no. 4 (2012): e50–e58, 10.1200/JOP.2011.000418. PMC3396830 23180999

[23] Y.‐C. T. Shih and C.‐R. Chien , “A Review of Cost Communication in Oncology: Patient Attitude, Provider Acceptance, and Outcome Assessment,” Cancer 123 (2017): 928–939. 27893929 10.1002/cncr.30423 PMC5339042

[24] S. Y. Zafar , F. Chino , P. A. Ubel , et al., “The Utility of Cost Discussions Between Patients With Cancer and Oncologists,” American Journal of Managed Care 21, no. 9 (2015): 607–615. 26618364

[25] S. F. Huntington , B. M. Weiss , D. T. Vogl , et al., “Financial Toxicity in Insured Patients With Multiple Myeloma: A Cross‐Sectional Pilot Study,” Lancet Haematology 2, no. 10 (2015): e408–e416. 26686042 10.1016/S2352-3026(15)00151-9

[26] J. Witte , K. Mehlis , B. Surmann , et al., “Methods for Measuring Financial Toxicity After Cancer Diagnosis and Treatment: A Systematic Review and Its Implications,” Annals of Oncology 30, no. 7 (2019): 1061–1070. 31046080 10.1093/annonc/mdz140 PMC6637374

[27] J. Jing , R. Feng , X. Zhang , M. Li , and J. Gao , “Financial Toxicity and Its Associated Patient and Cancer Factors Among Women With Breast Cancer: A Single‐Center Analysis of Low‐Middle Income Region in China,” Breast Cancer Research and Treatment 181, no. 2 (2020): 435–443, 10.1007/s10549-020-05632-3. 32306169

[28] K. Honda , B. Gyawali , M. Ando , et al., “Prospective Survey of Financial Toxicity Measured by the Comprehensive Score for Financial Toxicity in Japanese Patients With Cancer,” Journal of Global Oncology 5 (2019): 1–8. 10.1200/JGO.19.00003 PMC6550026 31070981

[29] B. Zeybek , E. Webster , N. Pogosian , et al., “Financial Toxicity in Patients With Gynecologic Malignancies: A Cross Sectional Study,” JGO: Journal of Gynecologic Oncology 2 (2021): e87. 10.3802/jgo.2021.32.e87 PMC8550931 34431257

[30] C. Friedes , S. Z. Hazell , W. Fu , et al., “Longitudinal Trends of Financial Toxicity in Patients With Lung Cancer: A Prospective Cohort Study,” JCO Oncology Practice 17 (2021): e1094–e1109, 10.1200/OP.20.00721. 33555936

[31] “Breast Cancer,” (2025), ESMO, https://www.esmo.org/guidelines/guidelines‐by‐topic/esmo‐clinical‐practice‐guidelines‐breast‐cancer/consensus‐recommendations‐breast‐cancer‐in‐young‐women‐bcy5.

[32] R. Lentz , A. B. Benson, 3rd , and S. Kircher , “Financial Toxicity in Cancer Care: Prevalence, Causes, Consequences, and Reduction Strategies,” Journal of Surgical Oncology 120 (2019): 85–92, 10.1002/jso.25374. 30650186

[33] S. Zhu and C. Lei , “Association Between Marital Status and All‐Cause Mortality of Patients With Metastatic Breast Cancer: A Population‐Based Study,” Scientific Reports 13 (2023): 9067. 37277464 10.1038/s41598-023-36139-8 PMC10241782

[34] G. Wiesemann , E. Anne Cox , D. S. Nichols , K. Ockerman , E. S. Satteson , and S. C. Sorice Virk , “Changes in Marital Status After Receiving the Diagnosis of Breast Versus Prostate Cancer: A Population‐Based Study,” Plastic and Reconstructive Surgery—Global Open 10, no. 10 Suppl (2022): 133–134. 10.1097/GOX.0000000000006494 PMC11845180 39989894

[35] G. Werutsky , M. Lopes , R. G. de Jesus , et al., “The Impact of a Breast Cancer Diagnosis on Marital Outcomes and Factors Associated With Divorce and Separation,” Revista Brasileira de Ginecologia e Obstetrícia 46 (2024): e‐rbgo60. 10.61622/rbgo/2024rbgo60 PMC11239212 38994465

[36] F. Mols , B. Tomalin , A. Pearce , B. Kaambwa , and B. Koczwara , “Financial Toxicity and Employment Status in Cancer Survivors. A Systematic Literature Review,” Support Care Cancer 28, no. 12 (2020): 5693–5708. 32865673 10.1007/s00520-020-05719-z PMC7686183

[37] S. M. W. Jones , “Financial Worry in People With Cancer: Relationship to Employment and Outcomes,” Psycho‐Oncology 31, no. 11 (2022): 1835–1842. 36109869 10.1002/pon.6034

[38] L. C. G. Landeiro , D. M. Gagliato , A. B. Fêde , et al., “Return to Work After Breast Cancer Diagnosis: An Observational Prospective Study in Brazil,” Cancer 124, no. 24 (2018): 4700–4710. 30329152 10.1002/cncr.31735

