# Supplementary material for: Financial Toxicity in Patients With Breast Cancer Treated at Private Medicine Institutions in Brazil
Source: Cancer Med. 2026 Mar 10;15(3):e71664. doi: 10.1002/cam4.71664 (PMC12976455; doi:10.1002/cam4.71664)
Supplement: Supplementary file 1 — Data S1: cam471664‐sup‐0001‐DataS1.docx. [file CAM4-15-e71664-s001.docx]

**Supplementary File**

S1**. Multiple comparisons among health insurance types (Tukey post hoc test)**

| **Comparison** | **Mean Difference** | **p-value** |
| --- | --- | --- |
| Private health plan (individual) vs. Individual membership-based plan | NS | >0.999 |
| Private health plan (individual) vs. Employer-sponsored plan (corporate) | NS | 0.350 |
| Private health plan (individual) vs. Other | NS | 0.999 |
| Private health plan (individual) vs. No health insurance | Higher | **0.002** |
| Individual membership-based plan vs. Employer-sponsored plan (corporate) | NS | 0.515 |
| Individual membership-based plan vs. Other | NS | 0.998 |
| Individual membership-based plan vs. No health insurance | Higher | **0.004** |
| Employer-sponsored plan (corporate) vs. Other | NS | 0.990 |
| Employer-sponsored plan (corporate) vs. No health insurance | Higher | **0.031** |
| Other vs. No health insurance | NS | 0.152 |

**Abbreviations:** NS = not significant.
**Statistical test:** Tukey’s post hoc test following ANOVA.
**Significance level:** p < 0.05.

S2**. Multiple comparisons among current employment status (Tukey post hoc test)**

| **Comparison** | **Mean Difference** | **p-value** |
| --- | --- | --- |
| Full-time employment vs. Part-time employment | NS | >0.999 |
| Full-time employment vs. Self-employed | NS | 0.081 |
| Full-time employment vs. Other | NS | 0.986 |
| Full-time employment vs. Homemaker | NS | 0.445 |
| Full-time employment vs. Sick leave/work disability | Higher | **<0.001** |
| Full-time employment vs. Retired | NS | 0.997 |
| Full-time employment vs. Unemployed | Higher | **<0.001** |
| Part-time employment vs. Self-employed | NS | 0.285 |
| Part-time employment vs. Other | NS | 0.996 |
| Part-time employment vs. Homemaker | NS | 0.585 |
| Part-time employment vs. Sick leave/work disability | Higher | **<0.001** |
| Part-time employment vs. Retired | NS | >0.999 |
| Part-time employment vs. Unemployed | Higher | **<0.001** |
| Self-employed vs. Other | NS | 0.572 |
| Self-employed vs. Homemaker | NS | >0.999 |
| Self-employed vs. Sick leave/work disability | Higher | **<0.001** |
| Self-employed vs. Retired | Lower | **0.034** |
| Self-employed vs. Unemployed | Higher | **<0.001** |
| Other vs. Homemaker | NS | 0.678 |
| Other vs. Sick leave/work disability | Higher | **0.001** |
| Other vs. Retired | NS | 0.998 |
| Other vs. Unemployed | Higher | **<0.001** |
| Homemaker vs. Sick leave/work disability | Higher | **0.001** |
| Homemaker vs. Retired | NS | 0.240 |
| Homemaker vs. Unemployed | Higher | **<0.001** |
| Sick leave/work disability vs. Retired | Lower | **<0.001** |
| Sick leave/work disability vs. Unemployed | NS | 0.163 |
| Retired vs. Unemployed | Higher | **<0.001** |

**Abbreviations:** NS = not significant.
**Statistical test:** Tukey’s post hoc test following ANOVA.
**Significance level:** p < 0.05.

S3. **Results of multiple comparisons (Tukey method) among racial groups**

| **Comparison** | Mean difference | **p-value** |
| --- | --- | --- |
| Asian vs. White | NS | 0.786 |
| Asian vs. Black | Higher | 0.007 |
| Asian vs. Other | NS | 0.151 |
| White vs. Black | Higher | <0.001 |
| White vs. Other | Higher | 0.024 |
| Black vs. Other | NS | 0.240 |

**Abbreviations:** NS = not significant.
**Statistical test:** Tukey’s post hoc test following ANOVA.
**Significance level:** p < 0.05.

S4. Results of multiple comparisons (Tukey method) for self-perceived health status

| **Comparison** | **Mean difference** | **P value** |
| --- | --- | --- |
| Excellent vs. Good | Higher | <0.001 |
| Excellent vs. Fair | Higher | <0.001 |
| Excellent vs. Poor | Higher | <0.001 |
| Good vs. Fair | Higher | <0.001 |
| Good vs. Poor | Higher | 0.005 |
| Fair vs. Poor | NS | 0.962 |

**Abbreviations:** NS = not significant.
**Statistical test:** Tukey’s post hoc test following ANOVA.
**Significance level:** p < 0.05.

S5. Results of multiple comparisons (Tukey method) for family income

| **Comparison** | **Mean Difference** | **p-value** |
| --- | --- | --- |
| < R$10,000/month vs. R$10,000–20,000/month | Lower | <0.001 |
| < R$10,000/month vs. R$20,000–50,000/month | Lower | <0.001 |
| < R$10,000/month vs. > R$50,000/month | Lower | <0.001 |
| R$10,000–20,000/month vs. R$20,000–50,000/month | Lower | 0.001 |
| R$10,000–20,000/month vs. > R$50,000/month | Lower | <0.001 |
| R$20,000–50,000/month vs. > R$50,000/month | Lower | 0.014 |

**Abbreviations:** NS = not significant.
**Statistical test:** Tukey’s post hoc test following ANOVA.
**Significance level:** p < 0.05.
